# Fano interference between collective modes in cuprate high-*T*_c_ superconductors

**DOI:** 10.1038/s41467-023-36787-4

**Published:** 2023-03-11

**Authors:** Hao Chu, Sergey Kovalev, Zi Xiao Wang, Lukas Schwarz, Tao Dong, Liwen Feng, Rafael Haenel, Min-Jae Kim, Parmida Shabestari, Le Phuong Hoang, Kedar Honasoge, Robert David Dawson, Daniel Putzky, Gideok Kim, Matteo Puviani, Min Chen, Nilesh Awari, Alexey N. Ponomaryov, Igor Ilyakov, Martin Bluschke, Fabio Boschini, Marta Zonno, Sergey Zhdanovich, Mengxing Na, Georg Christiani, Gennady Logvenov, David J. Jones, Andrea Damascelli, Matteo Minola, Bernhard Keimer, Dirk Manske, Nanlin Wang, Jan-Christoph Deinert, Stefan Kaiser

**Affiliations:** 1 Max Planck Institute for Solid State Research, Heisenbergstr. 1, 70569 Stuttgart, Germany; 2 Quantum Matter Institute, University of British Columbia, Vancouver, BC V6T 1Z4 Canada; 3 Department of Physics and Astronomy, University of British Columbia, Vancouver, BC V6T 1Z1 Canada; 4 4th Physics Institute, University of Stuttgart, 70569 Stuttgart, Germany; 5 Center for Ultrafast Science and Technology, School of Physics and Astronomy, Shanghai Jiao Tong University, Shanghai, 200240 China; 6 Helmholtz-Zentrum Dresden-Rossendorf, Bautzner Landstr. 400, 01328 Dresden, Germany; 7 International Center for Quantum Materials, School of Physics, Peking University, Beijing, 100871 China; 8 Institute of Solid State and Materials Physics, Technical University Dresden, 01062 Dresden, Germany; 9 Énergie Matériaux Télécommunications Research Centre, Institut National de la Recherche Scientifique, Varennes, Québec J3X 1S2 Canada; 10 Beijing Academy of Quantum Information Sciences, Beijing, 100913 China; 11 Present Address: Quantum Matter Institute, University of British Columbia, Vancouver, BC V6T 1Z4 Canada

**Keywords:** Superconducting properties and materials, Phase transitions and critical phenomena, Electronic properties and materials

## Abstract

Cuprate high-*T*_c_ superconductors are known for their intertwined interactions and the coexistence of competing orders. Uncovering experimental signatures of these interactions is often the first step in understanding their complex relations. A typical spectroscopic signature of the interaction between a discrete mode and a continuum of excitations is the Fano resonance/interference, characterized by the asymmetric light-scattering amplitude of the discrete mode as a function of the electromagnetic driving frequency. In this study, we report a new type of Fano resonance manifested by the nonlinear terahertz response of cuprate high-*T*_c_ superconductors, where we resolve both the amplitude and phase signatures of the Fano resonance. Our extensive hole-doping and magnetic field dependent investigation suggests that the Fano resonance may arise from an interplay between the superconducting fluctuations and the charge density wave fluctuations, prompting future studies to look more closely into their dynamical interactions.

## Abstract (web summary)

Cuprate superconductors are known for their intertwined interactions and coexistence of competing orders. Here, the authors observe a Fano resonance in the nonlinear THz response of La_2-x_Sr_x_CuO_4_, which may arise from a coupling between superconducting and charge-density-wave amplitude fluctuations.

## Introduction

**Fig. 1:**
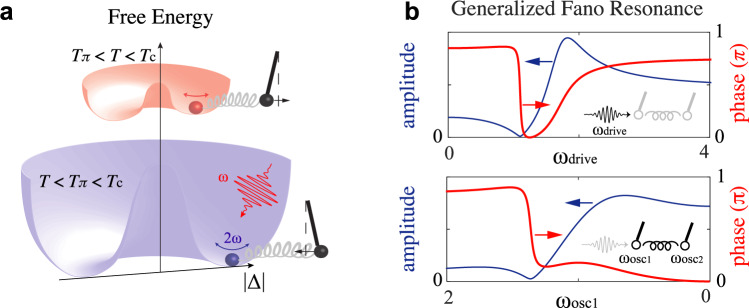
Nonlinear terahertz spectroscopy of *d*-wave superconductors. **a** An illustration of the amplitude oscillation of the superconducting order parameter (2*ω*) driven by electromagnetic radiation (*ω*) and its coupling to another collective mode (black pendulum) at temperatures above and below *T*_π_, i.e., the anti-resonance temperature. **b** The generalized Fano resonance model describes the interference between a driven damped (continuum) mode and an underdamped (discrete) mode, here represented as oscillators 1 and 2, respectively. The Fano resonance/interference is characterized by the asymmetrical line shape of the amplitude response (blue), and also by the negative π jump in the phase response (red), a.k.a. the anti-resonance. Upper panel: in typical spectroscopy experiments, the electromagnetic driving frequency (*ω*_drive_) is swept. Lower panel: in our experiment the driving frequency is fixed, but the resonance frequency of oscillator 1 (ω_osc1_) is swept. Here, oscillator 1 represents the superconducting fluctuations, whose energy scale is a function of temperature. By sweeping temperature from 0 towards *T*_c_, the energy of the superconducting fluctuations decreases from maximum to zero. We chose the direction of the *ω*_osc1_ axis as shown so that it corresponds to a temperature axis that increases to the right. Note, however, that sweeping temperature is not entirely equivalent to sweeping driving frequency, as additional parameters like the damping constant may depend on temperature. Details of the generalized Fano resonance model can be found in Supplementary Materials S8.

Interactions between the intrinsic degrees of freedom of a solid (e.g., charge, spin, orbital, and lattice) often lead to interesting physical properties or phenomena, such as BCS superconductivity, colossal magnetoresistance, and have been key to understanding and predicting novel phases of matter including topological insulators and unconventional superconductivity. Often, the signatures of these interactions manifest in the dynamical response of a system. For example, electron-phonon interaction may significantly renormalize the quasiparticle (electronic) dispersion, introducing kinks into the latter, which has been the subject of extensive research in the case of high-*T*_c_ superconductors^1–3^. The same interaction may also introduce discontinuities into the phonon dispersion, a well-known example being the Kohn anomaly arising from an electronic instability driven by Fermi surface nesting. When a discrete excitation interacts with a continuum of excitations, another kind of discontinuity could manifest in its light-scattering amplitude:^4,5^ an asymmetric amplitude profile may develop across the discrete mode, with one side showing a pronounced suppression (a.k.a. anti-resonance) (Fig. 1b). Known as the Fano resonance or the Fano interference, this quantum mechanical phenomenon is closely related to a classical counterpart, i.e., the driven coupled harmonic oscillators model^4^. In this classical model, if one oscillator is heavily damped (therefore analogous to a continuum of states) while the other is underdamped, the system would exhibit an anti-resonance where the amplitude of the driven oscillation goes through a minimum while its phase undergoes a negative π jump (opposite to the direction of a resonant phase jump) as a function of the driving frequency. In fact, the negative π phase jump is also intrinsic to a Fano resonance, although this particular phase signature has rarely been experimentally evidenced. In this work, we show that a careful extraction of the optical phase information from a terahertz-driven cuprate high-*T*_c_ superconductor allows us to uncover a Fano interference in its nonlinear terahertz response (Fig. 1a). In contrast, we do not find a similar effect in the conventional *s*-wave superconductors NbN. Such a contrast may arise from the presence of intertwined interactions in cuprate high-*T*_c_ superconductors.

A microwave/terahertz-driven superconductor has been known to exhibit strong nonlinearities across *T*_c_, particularly in terms of third harmonic generation (THG)^6–9^. Its origin has been suggested to relate to collective fluctuations inside a superconductor^10–13^, including the superconducting amplitude fluctuations (i.e., Higgs mode^14–18^), the Josephson plasma mode/the nonlinear Josephson current, and also the charge fluctuations (a.k.a. the BCS response, not to be confused with charge density wave fluctuations). In particular, for a conventional superconductor, the energy scale of the Higgs mode and the charge fluctuations follow a similar mean-field temperature dependence as 2Δ(*T*)^7,10,17^. They are expected to become resonantly driven when 2Δ(*T*) = 2*ω*, where ω is the frequency of the terahertz field. Therefore, it is difficult to distinguish between these different contributions based on the temperature dependence of THG. Nevertheless, for a BCS superconductor in the clean limit, different polarization dependence has been predicted for THG arising from each mechanism (see Supplementary Materials S6 for a detailed discussion)^10–13^, providing a potential way for distinguishing the origin of THG in an experiment.

One caveat about such an experiment is that dissimilar to most spectroscopy experiments in which the electromagnetic driving frequency is swept relative to an excitation fixed in energy, here the driving frequency is kept constant but the energy of the superconducting fluctuations, given by 2Δ(*T*), is swept by varying temperature (Fig.1b). For a reasonably small driving frequency ω, as we increase the temperature from 0 the superconducting fluctuations are first driven below resonance (i.e., 2*ω* < 2Δ_*T*~0_) and then above (i.e., 2*ω* > 2Δ_*T*~*T*c_ ~ 0). As illustrated in Fig. 1b, this means that as we heat up a superconductor we expect the THG phase to evolve positively across a resonance and negatively across an anti-resonance. For cuprate high-*T*_c_ superconductors where 2Δ, according to some experimental interpretations^19,20^, remains sizeable at *T*_c_, the resonance of the superconducting fluctuations might move beyond *T*_c_ for the small terahertz driving frequency of 0.7 THz used in our experiment. Here, the use of a narrow-band multi-cycle terahertz field generated from a superradiant high-field terahertz source TELBE allows us to precisely extract the phase information required for understanding any resonance or anti-resonance behavior of a periodically driven superconductor.

## Results

**Fig. 2:**
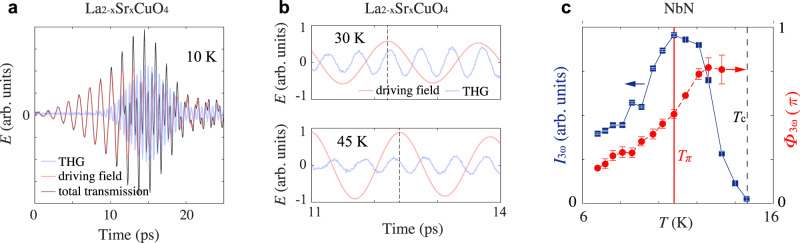
Third harmonic generation from terahertz-driven superconducting fluctuations. **a** Terahertz transmission from an overdoped superconducting La_2−*x*_Sr_*x*_CuO_4_ thin film as it is pumped by a 0.7 THz driving pulse. The latter drives the 2*ω* amplitude oscillation of the superconducting order parameter (see Supplementary Materials S5 for a clear experimental visualization of this 2*ω* oscillation from transient reflectivity measurements). This 2*ω* collective excitation scatters the driving photon, leading to third harmonic generation (THG) in transmission. The driving field (red) and the THG (blue) waveforms are extracted from the raw transmission data (black) using 1.4 THz Fourier lowpass and highpass filters. **b** Zoomed-in view of the transmitted driving field and THG from an optimally doped La_2−*x*_Sr_*x*_CuO_4_ thin film at 30 K and 45 K. The dotted line marks the crest of the driving field, which shifts in time due to the superconducting screening effect. It can be seen that THG does not remain in phase with the driving field at the two temperatures. **c** THG intensity (*I*_3ω_) and phase (Φ_3ω_, i.e., relative to the linear driving field phase) as a function of temperature from NbN. The peak in *I*_3ω_(*T*) and the continuous evolution of Φ_3ω_(*T*) across *T*_π_ indicate the resonance in THG. The error bars on *I*_3ω_ indicate the noise floors of the FFT power spectra; the error bars on Φ_3ω_ denote the uncertainties in fitting the THG phase and the linear drive phase.

Figure 2a shows the typical THG response of a superconducting cuprate thin film. In the raw transmission data, the THG response (3*ω* = 2.1 THz) is superposed on top of the transmitted linear driving field (*ω* = 0.7 THz), which can be separated from each other using Fourier lowpass and highpass filters. From their respective waveforms, we obtain their amplitude (*A*) and phase (*Φ*) by fitting them to the equation

1 $${{{{{\boldsymbol{E}}}}}}\left({{{{{\boldsymbol{t}}}}}}\right)={{{{{\boldsymbol{A}}}}}}\,{{{{{\mathbf{exp }}}}}}(-{\left({{{{{\boldsymbol{t}}}}}}-{{{{{{\boldsymbol{t}}}}}}}_{{{{{{\bf{0}}}}}}}\right)}^{{{{{{\bf{2}}}}}}}/{{{{{{\boldsymbol{c}}}}}}}^{{{{{{\bf{2}}}}}}}){{{{{\mathbf{sin }}}}}}\left({{{{{\boldsymbol{\omega }}}}}}\left({{{{{\boldsymbol{t}}}}}}-{{{{{{\boldsymbol{t}}}}}}}_{{{{{{\bf{0}}}}}}}\right)-{{{{{\boldsymbol{\Phi }}}}}}\right),$$

(only *A*, *Φ, c* are fitting parameters) which allows us to extract the relative phase between the THG response and the linear drive: ${\varPhi }_{3{{{{{\rm{\omega }}}}}}}-{3\varPhi }_{{{{{{\rm{\omega }}}}}}}$ (the factor 3 in front of *Φ*_ω_ accounts for the fact that one period of the linear drive is 3 times that of the THG). As an example, Fig. 2b shows the respective waveforms of the driving field and the THG at two representative temperatures. The phase of the linear transmission shifts between the two temperatures as a result of the superconducting screening effect^21^. The phase of THG does not simply follow the phase of the driving field: at 45 K its crest precedes that of the driving field while at 30 K its crest lags behind that of the driving field. The relative phase shift between the two, where the screening-induced phase shift of the driving field has been accounted for, reveals the intrinsic phase evolution of THG (See Supplementary Materials S3 and S4 for a detailed discussion about all contributions to phase shifts at *ω* and 3*ω*, which are nearly all accounted for by our analysis). It encodes important spectroscopic information about the superconducting fluctuations, such as their resonance or anti-resonance, across which a phase jump is expected.

**Fig. 3:**
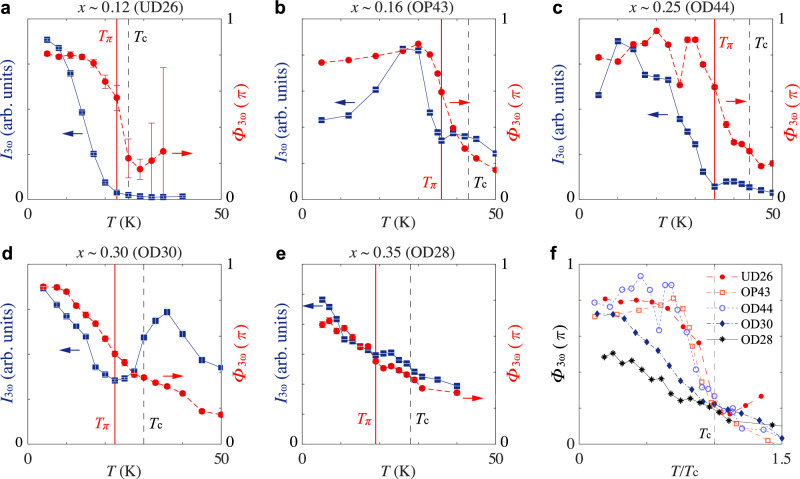
Doping dependence of Fano interference in La_2−*x*_Sr_*x*_CuO_4_. The Fano interference of the driven superconducting fluctuations is identified by the dip in THG intensity (*I*_3*ω*_) concomitant with the negative jump in THG phase (Φ_3*ω*_) at the temperature denoted by *T*_π_ (red solid line). The dip-peak feature in *I*_3*ω*_(*T*) near and above *T*_π_ gives the typical asymmetrical line shape associated with the Fano resonance/interference, compounded by an additional superconducting screening factor^9,21^. **a**–**e** Temperature dependence of *I*_3ω_ (blue squares) and Φ_3ω_ (red dots) in **a** *x* ~0.12 (UD26), **b** *x* ~0.16 (OP43), **c** *x* ~0.25 (OD44), **d** *x* ~0.30 (OD30), **e** *x* ~0.35 (OD28). The peak terahertz driving field strength used for these measurements are **a** ~10 kV/cm, **b** ~25 kV/cm, **c** ~25 kV/cm, **d** ~25 kV/cm, and **e** ~20 kV/cm. **f** Temperature dependence of Φ_3ω_ from all five samples plotted on the reduced temperature (*T*/*T*_c_) scale. The anti-resonance becomes increasingly broadened with hole-doping, indicating a broadening of the coupled mode on the overdoped side.

In the conventional *s*-wave superconductor NbN (*T*_c_ ~14 K, 2Δ_*T*=0_ ~5 meV), we observe a peak in THG intensity (*I*_3ω_) simultaneous with a positive evolution of its phase (Φ_3*ω*_) across *T* = 10.7 K (Fig. 2c), which indicates the resonance of the superconducting fluctuations under a periodic drive. The resonance temperature 10.7 K is roughly expected from the resonance condition 2*ω* = 2Δ(*T*), where the driving frequency *ω* used for this measurement is 0.5 THz (~2 meV) and 2Δ(*T*) follows a mean field-like temperature dependence. In the (optimally doped) cuprate high-*T*_c_ superconductors, we observe a slight positive evolution of Φ_3ω_ at low temperatures (Fig. 3b, see Supplementary Materials S11 for results from the bilayer cuprate DyBa_2_Cu_2_O_7−*x*_, where the positive phase evolution at low temperature is more apparent). The positive phase evolution, however, is interrupted at some higher temperature (*T*_π_) by an abrupt jump of nearly π in the negative direction. The phase jump is also accompanied by a dip in *I*_3*ω*_(*T*). Together, these features identify an anti-resonance, an essential part of Fano interference implying that the periodically driven superconducting fluctuations are coupled to another collective excitation.

To investigate the identity of the coupled mode, we look at the hole-doping dependence of the Fano interference first. We measured five La_2−*x*_Sr_*x*_CuO_4_ thin films grown under similar conditions spanning the underdoped, optimally-doped, and overdoped regimes. As shown in Fig. 3, the salient features of the anti-resonance can be identified at almost all doping levels. In particular, Φ_3ω_(*T*) is found to exhibit a more systematic evolution than *I*_3*ω*_(*T*) across different samples. This is because the temperature-dependent screening effect also causes a superconductor to reflect part of the incoming electromagnetic wave, leading to a variation of the driving field inside the sample as a function of temperature. Since *I*_3*ω*_ depends on both the intrinsic THG susceptibility (i.e., arising from the superconducting fluctuations) and the strength of the driving field, its evolution with temperature and hole-doping is not straightforward. In comparison, the relative phase difference Φ_3*ω*_ is free from the screening effect as explained above, therefore its evolution appears to be much more systematic. Based on the evolution of Φ_3*ω*_(*T*) in all five samples, it is clear that the anti-resonance is sharp in the underdoped and the optimally-doped samples and broadens with further hole-doping, which makes the dip in *I*_3*ω*_(*T*) more visible on the overdoped side. Within the generalized Fano resonance model, a broadening of the anti-resonance indicates that the coupled mode becomes more heavily damped. Overall, the doping dependence of the Fano interference indicates that the coupled mode is well-defined in the underdoped and up to the optimally doped samples, beyond which it becomes increasingly heavily damped at low temperatures.

**Fig. 4:**
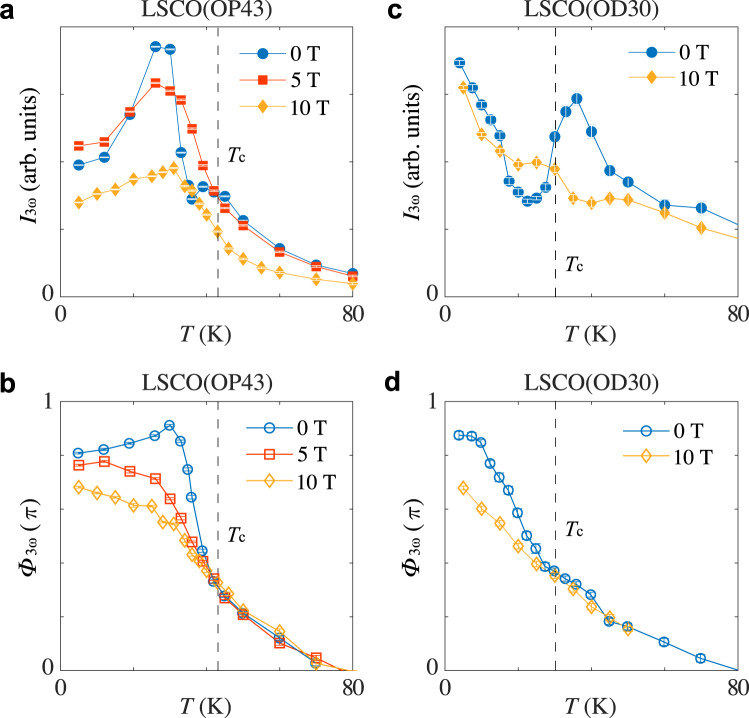
Magnetic field dependence of Fano interference in La_2-x_Sr_x_CuO_4_. **a**, **c** Temperature dependence of *I*_3*ω*_ with a magnetic field applied along the *c*-axis of **a** *x* ~0.16 (OP43), **c** *x* ~0.30 (OD30). **b**, **d** Corresponding temperature dependence of Φ_3*ω*_ from the two samples. The peak terahertz driving field strength used for these measurements is ~25 kV/cm. The anti-resonance becomes increasingly broadened by the magnetic field, indicating a broadening/softening of the coupled mode with a magnetic field.

In addition to the doping dependence, we also looked at the magnetic field dependence of the Fano interference. We measured the THG response of the *x* ~0.16 (OP43) and *x* ~0.30 (OD30) samples under a *c*-axis magnetic field. As shown in Fig. 4, a broadening of the anti-resonance is manifest in both the amplitude and phase response of THG: the dip feature in *I*_3*ω*_(*T*) disappears while the sharp jump in Φ_3*ω*_(*T*) widens. Together, these observations suggest that the coupled mode becomes heavily damped or softened upon the application of a magnetic field.

## Discussion

Below, we offer an interpretation of our results based on a hypothesized interaction between the terahertz-driven superconducting amplitude fluctuations (i.e., Higgs mode) and the CDW fluctuations, which are often found to coexist in the phase diagram of hole-doped cuprates (see Supplementary Materials S7 for a discussion about other possible scenarios for this coupled interaction). Such a hypothesis has also been put forth by previous pump–probe studies focusing on the CDW amplitude fluctuations in underdoped cuprates^22^, and is corroborated by recent reports of ubiquitous charge order fluctuations in a large part of the cuprate phase diagram overlapping with the superconducting dome and the pseudogap^23,24^. Note that this interpretation is predicated on assuming THG below *T*_c_ predominantly arises from the terahertz-driven Higgs oscillations^7,9,11^ (see Supplementary Materials S5 and S6 for a discussion about the different origins of THG in a superconductor).

To facilitate the discussion, we quickly summarize the current experimental understanding about the CDW/charge order in hole-doped cuprates: (1) the CDW correlation is strongest in underdoped cuprates and weakens with increasing hole-doping or temperature^25^ (note that recent studies nonetheless show evidence of CDW in overdoped LSCO and Tl2201^26,27^); (2) at ambient pressure the CDW correlation is generally two-dimensional and short-ranged at all temperatures and its growth upon cooling is pre-empted by the superconducting transition^28,29^. The second point here implies that any means that enhances the 2D CDW correlation, such as lowering temperature^28–31^ (i.e., until *T*_c_), applying a uniaxial pressure^32^ or magnetic field^33^, will bring the order parameter closer to its critical point and cause its collective excitations to soften.

Having this in mind, we recall that the doping dependence of the Fano interference indicates that the coupled mode is well-defined in the underdoped and optimally doped samples and becomes increasingly heavily damped in the overdoped samples. This is consistent with the doping dependence of the CDW correlation generally expected for cuprates. Second, a magnetic field is expected to suppress superconductivity macroscopically and enhance the CDW correlation^33^. As the CDW order parameter approaches the critical point, we expect its collective fluctuations to soften, which would also lead to a broadening of the anti-resonance. Here, we emphasize again that although the CDW correlation is strengthened by a magnetic field, its collective fluctuation is expected to soften because at ambient pressure it sits above the putative critical point inside a generic cuprate superconductor^28–30^. Finally, we note that a recent mean-field investigation of NbSe_2_ in which superconductivity and CDW coexist and dynamically interact indeed suggests that its THG response manifests a characteristic anti-resonance^34,35^, similar to our experimental observations above (see also Supplementary Materials S10).

While the interpretation of the Higgs-CDW amplitude mode interaction remains speculative at this stage, we note another interesting finding from the magnetic field dependence study: despite significant changes in *I*_3*ω*_(*T*) near *T*_π_ (i.e., the suppression of the Fano interference), the overall suppression of THG by the applied field is small up to 10 T in both samples above and below *T*_c_. This can be clearly seen for the OD30 sample in Fig. 3c but is more evident for both samples from the field-sweep measurements performed at constant temperatures (see Supplementary Materials S9). As the THG signal is sensitive to small fluctuations and drifts in the driving power (i.e., *I*_3*ω*_ ∝ *I*_*ω*_^3^) and a complete temperature-sweep measurement as shown in Fig. 3 takes many hours to acquire, the true field dependence of THG is more accurately reflected by the field-sweep results. The lack of field dependence in *I*_3*ω*_ away from *T*_π_ suggests that the length scale relevant to the THG process is more likely related to the superconducting coherence length (ξ_ab_ ~20–30 Å in cuprates) than the London penetration depth (λ_ab_ ~several hundred nm). The latter is known to increase in the presence of a magnetic field^36^, which would have led to a decrease in *I*_3*ω*_ at any given temperature. The coherence length, on the other hand, is more robust to external fields as it scales with the upper critical field *H*_c2_ as ξ_ab_ ∝ *H*_c2_^−^^1/2^, where *H*_c2_ is on the order of several tens of Teslas in these materials. Adopting such an interpretation would immediately suggest that the above-*T*_c_ THG, which is also observed in our studies, may arise from local preformed Cooper pairs. In comparison, in NbN THG drops to zero above *T*_c_, consistent with the absence of preformed Cooper pairs in conventional superconductors.

The above interpretation carries an important implication as in some of our measurements an above-*T*_c_ anti-resonance phase jump may also be inferred (Supplementary Materials S11), implying a non-vanishing interaction between preformed Cooper pairs and CDW above *T*_c_. Such an interpretation naturally reminds the intimate relationship between the two orders in the pseudogap region of the cuprate phase diagram. We note that a recent theoretical model, the pair density wave (PDW) model^37^, predicts a common origin of these two microscopic orders and an interference between Cooper pairs/CDW/PDW already above *T*_c_. To investigate the potential link between the PDW model and our findings, and to unlock the longstanding mysteries of high-*T*_c_ superconductors, we anticipate future studies to expand our results to greater details and shed further light on the nature of the Fano interference universally manifested by different families of cuprates.

## Methods

### Sample preparation

The La_2−*x*_Sr_*x*_CuO_4_ samples are grown by molecular beam epitaxy (MBE) method on LaSrAlO_4_ substrate. All samples are 40 nm thick. *T*_c_ is determined from mutual inductance measurements as shown in Supplementary Materials S2.

### Experimental method

The THG experiment is performed using the schematic shown in Supplementary Materials S1. The multicycle terahertz driving pulse is generated from the TELBE super-radiant undulator source at HZDR^38^. For the data presented in the main text, we use a driving frequency of 0.7 THz and place a 2.1 THz bandpass filter after the sample for filtering the THG response. The transmitted terahertz pulse is measured using electro-optical sampling inside a 2 mm ZnTe crystal and by using 100 fs gating pulses with a central wavelength of 800 nm. The accelerator-based driving pulse and the laser gating pulse have a timing jitter characterized by a standard deviation of ~20 fs. Synchronization is achieved through pulse-resolved detection as detailed in ref. ^39^.

## Supplementary information

Supplementary Information

## Data Availability

Data that support the findings of this study are available from the online repository 10.14278/rodare.1692.
