# Supplementary material for: Fano interference between collective modes in cuprate high-Tc superconductors
Source: Nat Commun. 2023 Mar 11;14:1343. doi: 10.1038/s41467-023-36787-4 (PMC10008591; doi:10.1038/s41467-023-36787-4)
Supplement: Supplementary file 1 — Supplementary Information [file 41467_2023_36787_MOESM1_ESM.pdf]

Supplementary Materials

Fano interference between collective modes in cuprate high-T_c superconductors

Hao Chu[1,2,3,4,5], Sergey Kovalev[6], Zi Xiao Wang[7], Lukas Schwarz[1], Tao Dong[7], Liwen Feng[1,4,8], Rafael Haenel[1,2,3], Min-Jae Kim[1,4,8], Parmida Shabestari[1,4], Le Phuong Hoang[1,4], Kedar Honasoge[1,4], Robert David Dawson[1], Daniel Putzky[1], Gideok Kim[1], Matteo Puviani[1], Min Chen[6], Nilesh Awari[6], Alexey N. Ponomaryov[6], Igor Ilyakov[6], Martin Bluschke[1,2,3], Fabio Boschini[9], Marta Zonno[1,2,3], Sergey Zhdanovich[2,3], Mengxing Na[2,3], Georg Christiani[1], Gennady Logvenov[1], David J. Jones[2,3], Andrea Damascelli[2,3], Matteo Minola[1], Bernhard Keimer[1], Dirk Manske[1], Nanlin Wang[7,10], Jan-Christoph Deinert[6], Stefan Kaiser[1,4,8]

[1] *Max Planck Institute for Solid State Research, Heisenbergstr. 1, 70569 Stuttgart, Germany*

[2] *Quantum Matter Institute, University of British Columbia, Vancouver, BC V6T 1Z4, Canada*

[3] *Department of Physics and Astronomy, University of British Columbia, BC V6T 1Z1, Canada*

[4] *4th Physics Institute, University of Stuttgart, 70569 Stuttgart, Germany*

[5] *Center for Ultrafast Science and Technology, School of Physics and Astronomy, Shanghai Jiao Tong University, Shanghai 200240, China*

[6] *Helmholtz-Zentrum Dresden-Rossendorf, Bautzner Landstr. 400, 01328 Dresden, Germany*

[7] *International Center for Quantum Materials, School of Physics, Peking University, Beijing 100871, China*

[8] *Institute of Solid State and Materials Physics, Technical University Dresden, 01062 Dresden, Germany*

[9] *Énergie Matériaux Télécommunications Research Centre, 1650 blvd Lionel-Boulet Varennes, Québec J3X 1S2, Canada*

[10] *Beijing Academy of Quantum Information Sciences, Beijing 100913, China*

S1. Experimental method

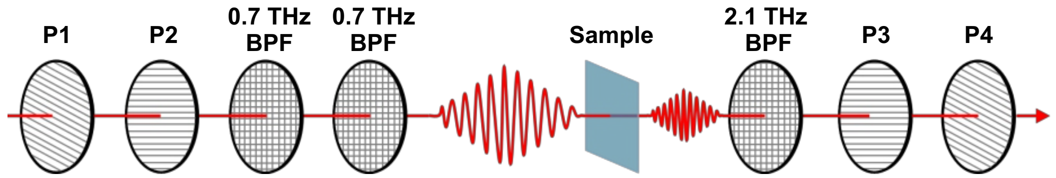

Figure S1 Experimental setup for measuring THz third harmonic generation. P1-P4: wire-grid polarizers. BPF: bandpass filter.

The THG experiment is performed using the schematic shown in Fig. S1. The multicycle terahertz driving pulse is generated from the TELBE super-radiant undulator source at HZDR[1]. For the data presented in the main text, we use a driving frequency of 0.7 THz and place a 2.1 THz bandpass filter after the sample for filtering the THG response. The transmitted terahertz pulse is measured using electro-optical sampling inside a 2 mm ZnTe crystal and by using 100 fs gating pulses with a central wavelength of 800 nm. The accelerator-based driving pulse and the laser gating pulse have a timing jitter characterized by a standard deviation of ~ 20 fs. Synchronization is achieved through pulse-resolved detection as detailed in ref(2).

To extract the relative phase between the THG response and the linear driving field, we fit a Gaussian-enveloped sinusoidal function, as detailed in ref(3), to both the transmitted linear driving field waveform and the THG waveform. From these fits, we obtain the phase of the driving field and the THG response for each temperature. We then take their difference (modulo 2π) while accounting for the fact that a 2π phase change for the driving field corresponds to a 6π phase change for the THG response. The result is double-checked by another method as detailed in the supplementary information of ref(3), where we first manually shift the transmitted driving field in time to overlap them in phase (i.e. getting rid of the temperature-dependent phase shift induced by the superconducting screening effect), and then apply the same time shifts to the THG waveforms at the corresponding temperatures. Then we extract the THG phase by fitting a Gaussian-enveloped sinusoidal function to the time-shifted THG waveforms. Both analysis methods yield very similar results, confirming the validity of the anti-resonance phase jump in the THG response

S2. Sample characterization

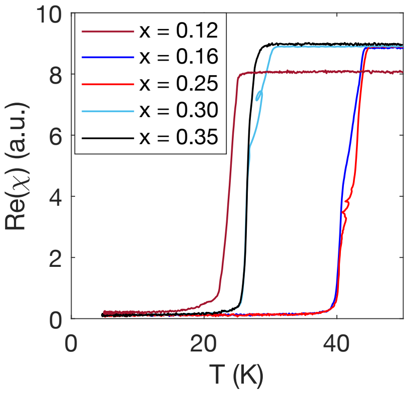

Figure S2 Magnetic susceptibility measurements of T_c on $La_{2-x}Sr_xCuO_4$. The superconducting transition temperature T_c is determined from the real part of the magnetic susceptibility χ. T_c is defined as the temperature for the initial drop in Re(χ).

The $La_{2-x}Sr_xCuO_4$ samples are grown by molecular beam epitaxy (MBE) method on $LaSrAlO_4$ substrate. All samples are 40 nm thick. The $DyBa_2Cu_2O_{7-x}$ samples (see **S11**) are also grown by MBE method on $(LaAlO_3)_{0.3}(Sr_2TaAlO_6)_{0.7}$ (LSAT) substrate. The OP 90 sample is 70 nm thick. The UD50 sample is 20 nm thick. T_c is determined from mutual inductance measurements as shown in Fig. S2 for the $La_{2-x}Sr_xCuO_4$ series.

Note that in thin $La_{2-x}Sr_xCuO_4$ epitaxial films grown by ozone assisted MBE the superconducting dome is between Sr-concentrations of x = 0.05 and x = 0.35, which differs from $La_{2-x}Sr_xCuO_4$ single crystals where dome is between x = 0.05 and x = 0.26. Some of the factors contributing to this difference include the fact that $La_{2-x}Sr_xCuO_4$ films are under compressive strain, and that oxygen vacancy concentration differs in the overdoped regime. Therefore, to be objective we report the sample doping level according to the Sr-concentration instead of the 'artificial' mobile carrier concentration p calculated from the experimentally measured T_c and established temperature-doping phase diagrams using the formula formula $T_c = A(p - p_{c1})(p_{c2} - p)$.

Note also that these samples exhibit different kinds of disorder: (a) non-stoichiometry: it depends on the stoichiometry control of MBE method for all doping levels. It is within error bar and is same for all samples. (b) distribution of dopants: the homogeneity of the dopants may be estimated from the sharpness of the superconducting transition from the above measurements. It can be seen that all the samples exhibit a reasonably sharp transition within 2 to 3 kelvins. Since the terahertz pulse used in this experiment has a small spot size (less than 1mm in diameter), the doping level may be considered homogeneous within the beam spot. (c) oxygen vacancies: they most favorably occupy the apical sites and their concentration increases with Sr-concentration. A detailed study of their evolution with doping can be found in ref(4).

S3. Extraction of relative phase between TH response and linear drive

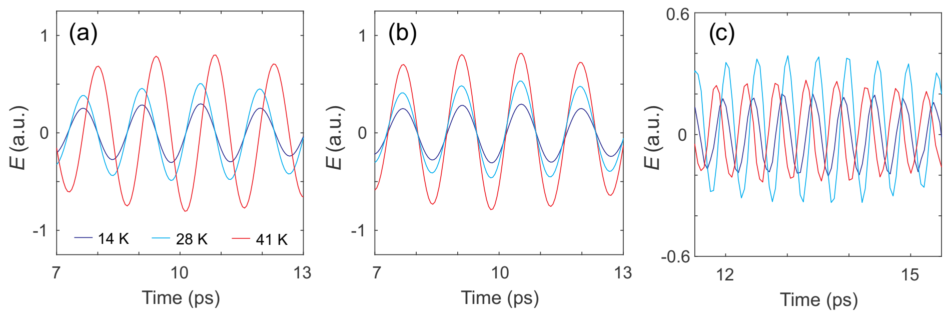

<u>Figure S3</u> Procedure for extracting the relative phase between TH response and linear drive. **a**, raw linear waveforms at a few representative temperatures below T_c in LSCO(OP45). **b**, linear waveforms aligned on top of each other after being shifted in time. **c**, TH waveforms at the same temperatures after applying the same time shift.

Here we demonstrate a different way of extracting the relative phase shift of THG not explicitly using the formula $\Phi_{3\omega}$ - $3\Phi_{\omega}$ in the main text. However, it follows the same basic principle: we correct the raw data (both linear transmission and THG) for the time shift induced on the linear driving pulse by the screening effect, and then extract the phase of the time-shifted THG waveforms.

As in the main text, the raw linear and TH waveforms are extracted from the raw transmitted waveforms using 1.4 THz FFT low pass and high pass filters. For example, a few extracted linear waveforms from an LSCO(OP45) sample are shown in Figure S3a. Due to the inductive response of superconductors below T_c, the linear wave experiences a phase shift across T_c on transmission through the superconducting thin film. This is also illustrated in Figure S3a. As a first step, we apply a time shift δt to the linear waveform at each temperature, so that their phases are all aligned with the lowest temperature waveform (Figure S3b). Then, we apply the same time shift δt to the corresponding TH waveform at each temperature. The resulting TH waveforms are shown in Figure S3c. Any phase shift between the TH waveforms in Figure S3c has to intrinsically come from the driven superconducting fluctuations because the phase shift in the linear drive has already been accounted for. To extract this relative TH phase, we then fitted the waveforms in Figure S3c to the same Gaussian-enveloped sinusoidal function as in the main text,

$$\boldsymbol{E_{\mathrm{TH}}(t) = A \exp\left(-(t-t_0)^2/c^2\right) \sin(\omega(t-t_0)-\Phi)},$$

where only A, c, $\boldsymbol{\Phi}$ are free fitting parameters. $\boldsymbol{\Phi}$ gives the relative TH phase with respect to the linear drive, which is equivalent to the THG phase extracted by using $\Phi_{3\omega}$ - $3\Phi_{\omega}$ as in the main text.

S4. Thin film transmission effects on THG relative phase

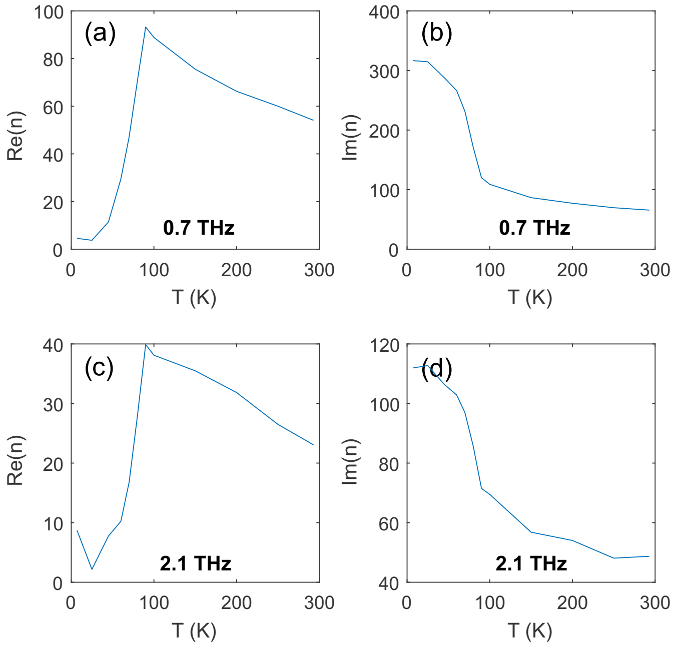

Figure S4 Refractive index of DyBCO(OP90) determined from ellipsometry measurements

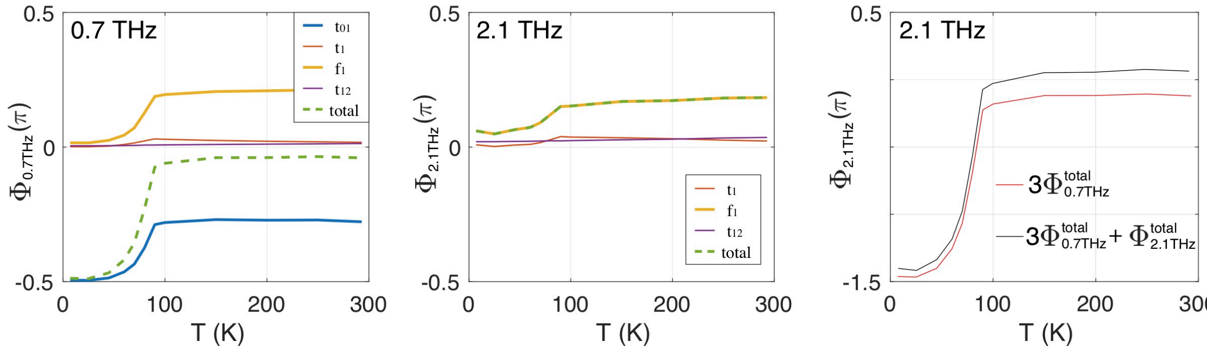

Figure S5 Phase shift accumulated during transmission through the vacuum-sample interface (t_{01}), through the sample (t_1), with Fabry-Perot effect taken into account (f_1), through the sample-substrate interface (t_{12}). Since phase shift is additive, the total phase shift is the sum of the phase shifts from t_{01}, f_1 and t_{12}. The calculation for 2.1 THz does not include the vacuum-sample interface (t_{01}) because THG is generated entirely within the sample. It can be seen that the phase shift acquired from the transmission effects (t_{01}, f_1 and t_{12}) at 2.1 THz is significantly smaller compared to that at 0.7 THz. The figure on the right shows the THG phase (black line) outside the sample had it been generated in-phase with the linear drive at all temperatures. That is, it simply follows the phase of the linear drive (multiplied by a factor of 3) plus the phase shift at 2.1 THz inherited from transmission effects (f_1, t_{12}). This small contribution (f_1, t_{12}) is not accounted for in the relative THG phase presented in the main text.

The refractive index of a superconductor changes dramatically during the superconducting transition. This affects the transmission of the 0.7 THz driving field and the 2.1 THz THG through the sample differently at different temperatures. Here we show that most of its effects on the THG relative phase has been implicitly accounted for in the formula $\Phi_{3\omega}$ - $3\Phi_{\omega}$.

Specifically, the temperature-dependent refractive index of the superconducting thin film makes the following transmission processes temperature-dependent: **a**) the complex transmission amplitude t_{01} across the vacuum(0)-sample(1) interface; **b**) the complex transmission amplitude t_1 of the electromagnetic wave through the thin film(1), which could include the Fabry-Perot effect f_1; **c**) the complex transmission amplitude t_{12} across the sample(1)-substrate(2) interface. Here, t_{01}, t_1, f_1, t_{12} can be all derived from the Fresnel equations. They are functions of the refractive indices of the vacuum, sample, and substrate.

In our experiment, the driving pulse sees the effect of t_{01}, t_1 and t_{12} whereas THG sees the effect of t_1 and t_{12} only. We use the experimentally determined refractive index (both the real and imaginary parts, n and k) of the bilayer cuprate DyBCO(OP90) (Fig. S4) to illustrate the effects of a temperature-dependent n_{ω}, k_{ω}, $n_{3\omega}$, $k_{3\omega}$ on the relative phase of THG[3].

As shown in Fig. S5, we find that the linear transmission accumulates phase shift mostly across the vacuum-sample interface (t_{01}), and from the Fabry-Perot effect (f_1) inside the film. In the formula $\Phi_{3\omega}$ - $3\Phi_{\omega}$, all the transmission effects on 0.7 THz (in the main text collectively referred to as the **'screening-induced phase shift'**) have been accounted for by subtracting the as-measured 0.7 THz phase shift from the as-measured 2.1 THz phase shift (illustrated in Fig.S5c). The only transmission effects which we didn't account for by the formula $\Phi_{3\omega}$ - $3\Phi_{\omega}$ is the transmission effects-induced phase shift of the 2.1 THz (f_1, t_{12}). Figure S5bc shows that below T_c this part is very small (between 0.05π and 0.1π) and varies slowly with temperature, which contrasts strongly with the sharp phase jump (particularly so in this DyBCO(OP90) sample) at T_π (Fig. S10). Therefore, we conclude that our observation of the sharp phase jump manifested in THG at T_π remains valid. A similar conclusion can be reached for variously doped LSCO thin films, where the temperature-induced change in the refractive index is smaller and hence gives an even smaller correction to the as-presented THG phase in the main text.

S5. Terahertz pump optical reflectivity probe results

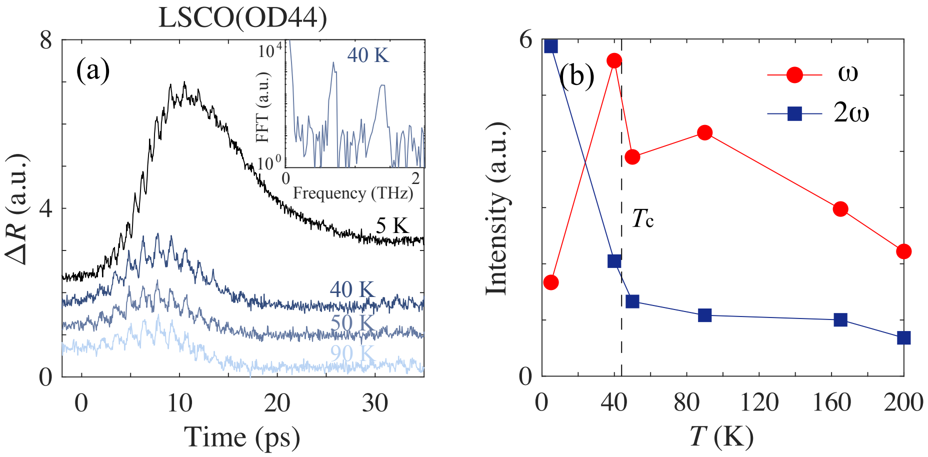

Figure S6 Terahertz-driven 2ω Higgs oscillations in transient reflectivity (a) Transient reflectivity at 800 nm wavelength measured as a function of terahertz pump-optical probe delay from an slightly overdoped LSCO thin film. The terahertz driving field is centered around 0.7 THz. The driven Higgs oscillations at 1.4 THz is manifest in both the time-domain reflectivity and its FFT (inset). **(b)** Temperature dependence of the intensity of the linear (ω) and Higgs (2ω) components, as extracted from FFT.

The driven Higgs oscillations have a typical spectroscopic signature in a terahertz pump optical reflectivity probe (TPOP) experiment, where a linear driving field at frequency ω induces the superconducting order parameter to oscillate at 2ω. This 2ω oscillation can be observed from transient optical reflectivity, as is done for an LSCO(OD44) thin film shown above. Fig. S6a shows that while the 0.7 THz driving pulse is on, optical reflectivity of the sample exhibits a clear 1.4 THz modulation on top of the 0.7 THz modulation. Interestingly, while this 2ω response enhances dramatically below T_c, it remains non-vanishing above T_c up to 200 K (Fig. S6b). The ω response, on the other hand, diverges near T_c, and could be associated with the pair-breaking process. The terahertz-driven current that gives rise to the ω response becomes screened below T_c by the superconducting transition, consistent with the drop of the ω amplitude at lower temperature. The presence of a sizeable Higgs response in our TPOP experiment is also in agreement with the experimental results of ref(5) on BSCCO single crystals.

S6. Proposed mechanisms of THG inside a superconductor

While driven Higgs oscillations have been proposed as a mechanism for generating THG inside a terahertz-driven superconductor[3,6-9], several additional mechanisms for THG has been considered in the context of a superconductor, including the pair-breaking/BCS process[10,11], and light scattering off two Josephson plasmons[12]. Regarding the relative intensity of the Higgs and BCS contributions to THG, theory suggests that the BCS contribution could dominate in clean *s*-wave superconductors[13], while the Higgs contribution still prevails in the dirty limit[11,13-16]. However, for *d*-wave cuprates superconductors, recent experimental and theoretical works[5,17] suggest that additional mechanisms can be responsible for a prevailing Higgs response, even without impurities.

The different mechanisms considered in these studies also lead to different polarization dependence in THG, which, depending on the level of disorder in the system, may be more or less pronounced[11,13,18]. THG originating from the BCS process as well as the two-plasmon process is expected to exhibit an anisotropic polarization dependence in agreement with the symmetry of the underlying electronic structure, and is allowed in both parallel and cross-polarized channels (as an example, see ref(19) for optical THG anisotropy from Sr_2IrO_4, a compound with very similar symmetry and electronic structure to La_2CuO_4). In contrast, THG originating from the Higgs mode in clean superconductors is expected to exhibit an isotropic polarization dependence, and is polarized parallel to the incoming driving field. As shown in our previous study (3), THG from cuprate thin films is characterized by a dominant isotropic component, with a small anisotropic component potentially on top. In the present study, we have also verified that THG in the cross-polarized channel is at least an order of magnitude weaker compared to the parallel-polarized channel. Together, these results are consistent with a dominant Higgs contribution to THG in our experiment. The great enhancement of THG below T_c as a signature of the growing Higgs response (due to the growing superconducting OP) is also testified by the pronounced 2ω component in the terahertz pump optical reflectivity probe (TPOP) measurement (**S5**) below T_c. In addition, the persistence of the 2ω component above T_c in these TPOP measurements is also in line with the non-vanishing THG above T_c, suggesting a common physical origin of the two types of nonequilibrium signals, namely the driven Higgs oscillations.

An additional piece of evidence for the dominant Higgs contribution to THG in our experiment comes from the observation of the Fano resonance/interference itself. As our mean field model (**S10**) indicates, light scattering off an independent collective mode (e.g. the Higgs mode, or the CDW amplitude mode) gives rise to a single resonance in THG accompanied by a positive phase jump. In order to produce the experimentally observed anti-resonance/Fano interference, theory requires two collective modes to interact with each other. Among the proposed THG mechanisms as discussed above, this requires an interaction between the Higgs mode, the Josephson plasmon, and the CDW amplitude mode as considered in this work. Based on the present understanding of

cuprates, an interaction between the Higgs mode and the CDW is more plausible than other combinations: CDW-plasmon interaction (which doesn't invoke the Higgs mode as a source of THG) for example. This then suggests that the main microscopic processes behind THG, at least in our studies, are described by the Feynman diagrams as shown Fig. S8g, where the Higgs mode is a key ingredient.

S7. A discussion about the other candidates as the coupled mode

In the main text, we mentioned that in addition to CDW fluctuations, antiferromagnetic spin fluctuations, an oxygen-buckling B_{1g} phonon, and the Josephson plasma mode are other collective modes often discussed in the context of cuprate high-T_c superconductors. Here, we briefly discuss why we don't consider them as the primary candidate for the coupled mode.

First of all, we may quickly rule out the oxygen-buckling B_{1g} phonon, which has an energy around 10 THz and therefore doesn't come close to our driving frequency. We may also rule out the Josephson plasma mode (or JPR: Josephson plasma resonance), for the reasons discussed in S6 (i.e. lack of anisotropy in THG polarization dependence and lack of experimental evidence supporting the Higgs-JPR coupling picture).

Now, neutron scattering experiments show that the low-energy spin fluctuations become gapped out below the superconducting transition. Its spectral weight is transferred to the high energy part which comprises a prominent underdamped/coherent mode at the antiferromagnetic wavevector with an energy slightly below 2Δ [20]. The latter is known as the magnetic resonance mode and onsets above the superconducting transition. It is considered as a candidate for the strongly coupled boson behind the nodal kink observed in ARPES studies. The low energy spin fluctuations, on the other hand, can be enhanced in superconducting samples by introducing a *c*-axis magnetic field[21]. In optimally-doped LSCO single crystals, they re-appear with a characteristic energy around 4 meV at an applied field of 7.5 T. This doesn't seem consistent with the 'softening' of the coupled mode inferred from our magnetic field dependence study. The magnetic resonance mode, in comparison, exhibits a weak temperature dependence across T_c [20]. Its characteristic frequency lies also far above our terahertz driving frequency. Therefore, an anti-resonance is unlikely to be seen in our experiment even if a coupling exists between the Higgs mode and the magnetic resonance mode. For these reasons, neither the low energy nor high energy spin fluctuations fits our experimental results.

S8. Generalized Fano resonance model

In our previous study[3], we have identified the anti-resonance as a result of coupling to an additional collective mode based on a driven coupled harmonic oscillators model. This model provides the basis for the generalized Fano resonance model: the well-known formula of the Fano resonance is an approximation of this model near the coupled discrete/underdamped mode[22]. In the following, we recapitulate the essence of ref(22) and show that the two models are essentially equivalent.

The equations of motion of a coupled oscillators system in which one is driven periodically are given by

$$\ddot{x}_1 + \gamma_1 \dot{x}_1 + \omega_1^2 x_1 + \upsilon_{12} x_2 = F_0 \cos(\omega t)\,,$$

$$\ddot{x}_2 + \gamma_2 \dot{x}_2 + \omega_2^2 x_2 + \upsilon_{12} x_2 = 0\,,$$

where ω_1, γ_1, ω_2, γ_2 are the frequency and damping constant of oscillators 1 and 2 respectively. ν_{12} is the coupling constant, F_0 is the driving force. x_1 is found to be

$$x_1 = \frac{\omega_2^2 - \omega^2 + i\gamma_2\omega}{(\omega_1^2 - \omega^2 + i\gamma_1\omega)(\omega_2^2 - \omega^2 + i\gamma_2\omega) - \upsilon_{12}^2} F_0 \cos(\omega t)$$

Alternatively, we can find the new eigenmodes of the coupled system as

$$\omega_a^2 = \omega_1^2 - \frac{\nu_{12}^2}{\omega_2^2 - \omega_1^2}\,,$$

$$\omega_b^2 = \omega_2^2 + \frac{\nu_{12}^2}{\omega_2^2 - \omega_1^2}\,.$$

Defining $\tilde{\epsilon} \equiv \omega^2 - \omega_b^2 = (\omega + \omega_b)(\omega - \omega_b)$, which is proportional to $(\omega - \omega_b)$ in the neighborhood of $\tilde{\epsilon} \sim 0$, i.e. $\omega \sim \omega_b$, we can define the reduced energy ϵ for the system

$$\epsilon \equiv \frac{1}{\gamma_1\omega_2} \frac{(\omega_2^2 - \omega_1^2)^2}{\nu_{12}^2} \tilde{\epsilon}$$

Assume $\gamma_2 = 0$, we can transform the numerator of x_1 near ω_b (i.e. $\omega \sim \omega_b$ or $\tilde{\epsilon} \sim 0$) as

$$\omega_2^2 - \omega^2 = -\tilde{\epsilon} - \frac{\nu_{12}^2}{\omega_2^2 - \omega_1^2}$$

and the denominator as

$$(\omega_1^2 - \omega^2 + i\gamma_1\omega)(\omega_2^2 - \omega^2) - \upsilon_{12}^2$$

$$= (\omega_2^2 - \omega_1^2)\tilde{\epsilon} + (\omega^2 - \omega_2^2 - i\gamma_1\omega)\tilde{\epsilon} + (\omega^2 - \omega_2^2 - i\gamma_1\omega)\frac{\nu_{12}^2}{\omega_2^2 - \omega_1^2}$$

$$\approx (\omega_2^2 - \omega_1^2)\tilde{\epsilon} - i\gamma_1\omega \frac{\nu_{12}^2}{\omega_2^2 - \omega_1^2}$$

Plugging these expressions back to x_1, using the definition of ϵ above we have

$$x_1 = \frac{-\tilde{\epsilon} - \frac{v_{12}^2}{\omega_2^2 - \omega_1^2}}{(\omega_2^2 - \omega_1^2)\tilde{\epsilon} - i\gamma_1\omega\frac{v_{12}^2}{\omega_2^2 - \omega_1^2}} F_0 \cos(\omega t) = \frac{\gamma_1\omega_2 \frac{v_{12}^2}{(\omega_2^2 - \omega_1^2)^2}(-\epsilon - \frac{\omega_2^2 - \omega_1^2}{\gamma_1\omega_2})}{\gamma_1\omega_2 \frac{v_{12}^2}{(\omega_2^2 - \omega_1^2)^2}(\epsilon - i)} \frac{F_0 \cos(\omega t)}{\omega_2^2 - \omega_1^2}$$

$$= -\frac{(\epsilon + q)}{(\epsilon - i)} \frac{F_0 \cos(\omega t)}{\omega_2^2 - \omega_1^2}, \qquad \text{where } q \equiv \frac{\omega_2^2 - \omega_1^2}{\gamma_1\omega_2}.$$

Here we see that the amplitude square (i.e. intensity) of x_1 becomes

$$|x_1|^2 \sim \frac{(\epsilon + q)^2}{\epsilon^2 + 1} \frac{{F_0}^2}{(\omega_2^2 - \omega_1^2)^2} \propto \frac{(\epsilon + q)^2}{\epsilon^2 + 1}$$

recovering the familiar Fano resonance formula.

For plotting Fig. 1b of the main text, we have used the following parameters and assumptions. For the upper figure, we used $\omega_1 = 1$, $\omega_2 = 1.1$, $\gamma_1 = 0.2$, $\gamma_2 = 0.02$ (introduced here to give a finite width to the anti-resonance), $v_{12} = 0.5$ while sweeping the driving frequency ω. For the lower figure, $\omega = 1.5$ (fixed driving frequency), $v_{12} = 0.5$. ω_1 is swept while keeping the damping constant as a fixed ratio $\gamma_1 = 0.2\omega_1$. ω_2 and γ_2 are also kept as a fixed ratio of ω_1: $\omega_2 = 1.1\omega_1$ and $\gamma_2 = 0.02\omega_1$. That is, the ratio between ω_1, γ_1, ω_2, γ_2 are identical across the upper and lower figures (for keeping the model simplistic and avoid making particular assumptions about the temperature-dependent softening, scattering rate, etc.)

S9. Magnetic field-sweep results

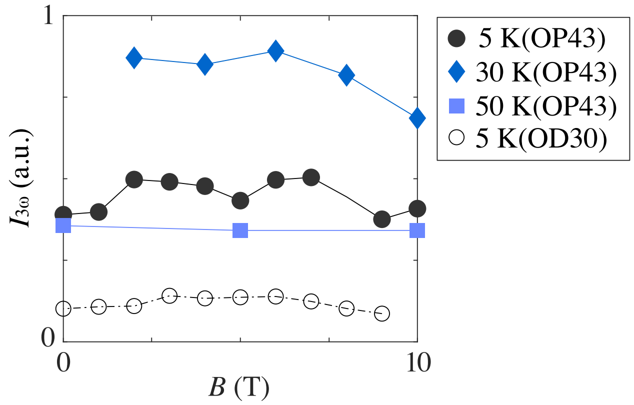

Figure S7 THG intensity as a function of magnetic field at constant temperatures

We measured the THG response of the $x \sim 0.16$ (OP43) and $x \sim 0.30$ (OD30) sample as a function of magnetic field (along *c*-axis) at constant temperatures. It can be seen that away from T_π (~ 36 K for $x \sim 0.16$ and ~ 22 K for $x \sim 0.30$), the THG intensity changes very weakly with magnetic field up to 10 Tesla. Each data point shown here takes about 15 minutes to acquire, therefore one field-sweep can be done within one to two hours. Therefore, this measurement is less prone to fluctuations or drifts in the driving pulse power, as compared to the temperature-sweep results shown in the main text.

S10. Microscopic model for the interplay between superconductivity and CDW

To model the interplay between superconductivity and a charge-density wave (CDW) on a microscopic level, we make use of a mean-field theory as it was established e.g. in ref(23) to describe the coexistence of superconductivity and a phonon-mediated CDW in $NbSe_2$. This model does not incorporate any cuprate specific details such that a quantitative agreement with the experiment cannot be expected. It rather serves as a minimal model describing the basic interplay and coupling mechanism between two collective modes.

We start from the following BCS Hamiltonian coupled to light and include a CDW phonon

$$H(t) = \sum_{\boldsymbol{k},\sigma} \epsilon_{\boldsymbol{k}} c^{\dagger}_{\boldsymbol{k},\sigma} c_{\boldsymbol{k},\sigma} - V \sum_{\boldsymbol{k},\boldsymbol{k}'} c^{\dagger}_{\boldsymbol{k},\uparrow} c^{\dagger}_{-\boldsymbol{k},\downarrow} c_{-\boldsymbol{k}',\downarrow} c_{\boldsymbol{k}',\uparrow} + \sum_{\boldsymbol{q}=\pm\boldsymbol{Q}} \omega_{\boldsymbol{q}} b^{\dagger}_{\boldsymbol{q}} b_{\boldsymbol{q}}$$
$$+ g \sum_{\boldsymbol{k},\boldsymbol{q}=\pm\boldsymbol{Q},\sigma} g_{\boldsymbol{k}} c^{\dagger}_{\boldsymbol{k}+\boldsymbol{q},\sigma} c_{\boldsymbol{k},\sigma} \left(b_{\boldsymbol{q}} + b^{\dagger}_{-\boldsymbol{q}}\right) + \frac{1}{2} \sum_{\boldsymbol{k},\sigma} \sum_{i,j} \partial^2_{ij} \epsilon_{\boldsymbol{k}} \boldsymbol{A}_i(t) \boldsymbol{A}_j(t) c^{\dagger}_{\boldsymbol{k},\sigma} c_{\boldsymbol{k},\sigma}$$

Hereby, $\epsilon_{\boldsymbol{k}} = \xi_{\boldsymbol{k}} - \epsilon_F$ is the electron band dispersion measured relative to the Fermi level ϵ_F, $c^{\dagger}_{\boldsymbol{k}}$ and $c_{\boldsymbol{k}}$ the electron creation and annihilation operators and V an s-wave pairing interaction. Furthermore, we consider a single phonon with wave vector $\boldsymbol{Q}$, which will be responsible for creating the CDW. The corresponding phonon creation and annihilation operators are $b^{\dagger}_{\boldsymbol{Q}}$ and $b_{\boldsymbol{Q}}$ and the phonon energy is $\omega_{\mathbf{Q}}$. The phonon interacts with the electrons via the electron-phonon coupling strength $g \cdot g_{\boldsymbol{k}}$, where g is the strength and $g_{\boldsymbol{k}}$ the symmetry of the interaction. The coupling to an external gauge field $\boldsymbol{A}$(t) is obtained via minimal coupling $\epsilon_{\boldsymbol{k}} \to \epsilon_{\boldsymbol{k}-\boldsymbol{A}(t)}$, where we expand in orders of $\boldsymbol{A}$(t)

$$\epsilon_{\boldsymbol{k}-\boldsymbol{A}(t)} = \epsilon_{\boldsymbol{k}} - \sum_{i} \partial_i \epsilon_{\boldsymbol{k}} \boldsymbol{A}_i(t) + \frac{1}{2} \sum_{i,j} \partial^2_{ij} \epsilon_{\boldsymbol{k}} \boldsymbol{A}_i(t) \boldsymbol{A}_j(t) + O(A(t)^3)$$

using $\partial_i = \partial_{\boldsymbol{k}_i}$ and $\partial^2_{ij} = \partial_{\boldsymbol{k}_i} \partial_{\boldsymbol{k}_j}$ for shorthand notation. As the system is parity symmetric, the linear coupling in $\boldsymbol{A}$ vanishes and only the diamagnetic coupling remains.

To simplify the calculation, we will consider a tight-binding dispersion on a square lattice at half-filling $\epsilon_{\boldsymbol{k}} = -2t\,(\cos k_x + \cos k_y)$ such that the system has perfect particle-hole symmetry. Thus, a CDW vector $\boldsymbol{Q} = (\pi, \pi)$ yields perfect nesting and the CDW is commensurate with $\boldsymbol{k} + 2\boldsymbol{Q} \triangleq \mathbf{k}$, i.e. $\epsilon_{\boldsymbol{k}+\boldsymbol{Q}} = -\epsilon_{\boldsymbol{k}}$ or $\epsilon_{\boldsymbol{k}+2\boldsymbol{Q}} = \epsilon_{\boldsymbol{k}}$. For the symmetry of the electron-phonon coupling we assume an anisotropic s-wave with $g_{\boldsymbol{k}} = |\cos k_x - \cos k_y|$.

We develop an effective field theory description with the partition function

$$\mathcal{Z} = \int \mathcal{D}(c^{\dagger}, c,\, b^{\dagger}, b) e^{-S(c^{\dagger}, c,\, b^{\dagger}, b)}\,,$$

where the action S in the Matsubara formalism is given by

$$S(c^\dagger, c, b^\dagger, b) = \int_0^\beta d\tau \left(\sum_{\boldsymbol{k},\sigma} c^\dagger_{\boldsymbol{k},\sigma}(\tau)\partial_\tau c_{\boldsymbol{k},\sigma}(\tau) + \sum_{\boldsymbol{q}=\pm\boldsymbol{Q}} b^\dagger_{\boldsymbol{q}}(\tau)\partial_\tau b_{\boldsymbol{q}}(\tau) + H(\tau) \right).$$

Decoupling the interaction with a Hubbard-Stratonovich transformation and rewriting the system with the four-component Nambu-spinor $\psi^\dagger_{\boldsymbol{k}} = \left(c^\dagger_{\boldsymbol{k},\uparrow}, c^\dagger_{\boldsymbol{k}+\boldsymbol{Q},\uparrow}, c_{-\boldsymbol{k},\downarrow}, c_{-(\boldsymbol{k}+\boldsymbol{Q}),\downarrow}\right)$, we obtain in frequency representation (see also ref(23))

$$S(\psi^\dagger, \psi, \delta\Delta, \delta D) = \beta\frac{\Delta^2}{V} + \beta\frac{D^2}{W} + \frac{1}{\beta}\sum_{i\omega_m} \delta\Delta(i\omega_m)\frac{1}{V}\delta\Delta(-i\omega_m) - \frac{1}{g^2}\frac{1}{\beta}\sum_{i\omega_m} \delta D(i\omega_m)P_0^{-1}(i\omega_m)\delta D(-i\omega_m)$$
$$-\frac{1}{\beta^2}\sum_{i\omega_m, i\omega_n}\sum_{\boldsymbol{k},\boldsymbol{k}'} \psi^\dagger_{\boldsymbol{k}}(i\omega_m)G^{-1}(\boldsymbol{k},\boldsymbol{k}', i\omega_m, i\omega_n)\psi^\dagger_{\boldsymbol{k}'}(i\omega_n)$$

with the bare phonon propagator

$$P_0^{-1}(i\omega_m) = -\frac{\omega_{\boldsymbol{Q}}^2 - (i\omega_m)^2}{2\omega_{\boldsymbol{Q}}}$$

and the BCS Green's function with Pauli matrices τ_i

$$G^{-1}(\boldsymbol{k},\boldsymbol{k}', i\omega_m, i\omega_n) = G_0^{-1}(\boldsymbol{k},\boldsymbol{k}', i\omega_m, i\omega_n) - \Sigma(\boldsymbol{k},\boldsymbol{k}', i\omega_m - i\omega_n),$$

$$G_0^{-1}(\boldsymbol{k},\boldsymbol{k}', i\omega_m, i\omega_n) = \left(i\omega_m\tau_0 \otimes \tau_0 - h^{(0)}_{\boldsymbol{k}}\right)\beta\delta_{\boldsymbol{k},\boldsymbol{k}'}\delta_{i\omega_m, i\omega_n} = G_0^{-1}(\boldsymbol{k}, i\omega_m)\beta\delta_{\boldsymbol{k},\boldsymbol{k}'}\delta_{i\omega_m, i\omega_n},$$

$$\Sigma(\boldsymbol{k},\boldsymbol{k}', i\omega_m - i\omega_n) = h^{(1)}_{\boldsymbol{k}}(i\omega_m - i\omega_n)\delta_{\boldsymbol{k},\boldsymbol{k}'}.$$

Here the cross product of Pauli matrices $\tau_i \otimes \tau_j$, $i, j = 0, \dots 3$, lies in the 4-component SC-CDW extended Nambu space: in particular, the first matrix refers to the SC subspace, while the second one acts in the CDW subspace. Hereby, the mean-field Hamiltonian $H(t) = \sum_{\boldsymbol{k}} \psi^\dagger_{\boldsymbol{k}} h_{\boldsymbol{k}}(t)\psi_{\boldsymbol{k}}$ in Nambu basis is defined as

$$h_{\boldsymbol{k}}(t) = h^{(0)}_{\boldsymbol{k}} + h^{(1)}_{\boldsymbol{k}}(t),$$

$$h^{(0)}_{\boldsymbol{k}} = \epsilon_{\boldsymbol{k}}\tau_3 \otimes \tau_3 - \Delta\tau_1 \otimes \tau_0 - Dg_{\boldsymbol{k}}\tau_3 \otimes \tau_1,$$

$$h^{(1)}_{\boldsymbol{k}}(t) = \frac{1}{2}\sum_{i,j} \partial^2_{ij}\epsilon_{\boldsymbol{k}} A_i(t)A_j(t)\,\tau_3 \otimes \tau_3 - \delta\Delta(t)\tau_1 \otimes \tau_0 - g_{\boldsymbol{k}}\delta D(t)\tau_3 \otimes \tau_1.$$

In these expressions, we introduce the superconducting order parameter Δ and CDW order parameter $D_{\boldsymbol{k}} = Dg_{\boldsymbol{k}}$ defined as

$$\Delta = 2V \sum_{\boldsymbol{k}} \langle c_{-\boldsymbol{k},\downarrow} | c_{\boldsymbol{k},\uparrow} \rangle = V \sum_{\boldsymbol{k}} \frac{\Delta}{E_{\boldsymbol{k}}} \tanh\left(\frac{\beta E_{\boldsymbol{k}}}{2}\right) ,$$

$$D \; = W \sum_{\boldsymbol{k},\sigma} g_{\boldsymbol{k}} \langle c^{\dagger}_{\boldsymbol{k},\sigma} c_{\boldsymbol{k}+\boldsymbol{Q},\sigma} \rangle = W \sum_{\boldsymbol{k}} g_{\boldsymbol{k}}^2 \frac{D}{E_{\boldsymbol{k}}} \tanh\left(\frac{\beta E_{\boldsymbol{k}}}{2}\right) ,$$

with $W = 4g^2/\omega_{\mathbf{Q}}$, where we allow amplitude fluctuations $\Delta(t) = \Delta + \delta\Delta(t)$ and $D(t) = D + \delta D(t)$. Please note, as the system has perfect particle-hole symmetry, phase fluctuations can be neglected as any coupling to long-range Coulomb interaction is identical zero. A diagonalization of the Hamiltonian yields the quasiparticle energy $E_{\boldsymbol{k}} = \sqrt{\epsilon_{\boldsymbol{k}}^2 + \Delta^2 + |D_{\boldsymbol{k}}|^2}$.

Next, we integrate out the fermions using $\int \mathcal{D}(\psi^\dagger, \psi) e^{-\psi^\dagger X \psi} = e^{\mathrm{tr}\,(\ln X)}$, where the trace includes the momentum and Matsubara sum. The logarithm can be expanded for small Σ via $\mathrm{tr}\ln(-G^{-1}) = \mathrm{tr}\ln(-G_0^{-1}) - \mathrm{tr}\sum_{n=1}^{\infty} \frac{(G_0\Sigma)^n}{n}$ and the action is split into a mean-field part and a fluctuation part

$$S(\delta\Delta, \delta D) = S_{\mathrm{MF}} + S_{\mathrm{FL}}(\delta\Delta, \delta D)$$

with

$$S_{\mathrm{MF}} = \beta \frac{\Delta^2}{V} + \beta \frac{D^2}{W} - \; \mathrm{tr}\ln(-G_0^{-1}) \; ,$$

$$S_{\mathrm{FL}}(\delta\Delta, \delta D) = \frac{1}{\beta} \sum_{i\omega_m} \delta\Delta(i\omega_m) \frac{1}{V} \delta\Delta(-i\omega_m) - \frac{1}{g^2}\frac{1}{\beta} \sum_{i\omega_m} \delta D(i\omega_m) P_0^{-1}(i\omega_m) \delta D(-i\omega_m) + \mathrm{tr} \sum_{n=1}^{\infty} \frac{(G_0\Sigma)^n}{n} \; .$$

As we are interested in the THG signal $j^{(3)} = \delta S/\delta A \propto A^3$, the terms in the action with power of A^4 are relevant. Thus, we consider the second order term in the sum of the trace $\frac{1}{2}\mathrm{tr}(G_0\Sigma G_0\Sigma)$. Neglecting the bare response, it follows for the fourth order action

$$S^{(4)}(\phi) = \frac{1}{2}\frac{1}{\beta} \sum_{i\omega_m} [\phi^\top(-i\omega_m) M(i\omega_m) \phi(i\omega_m) + \phi^\top(-i\omega_m) b(i\omega_m) + b^\top(-i\omega_m) \phi(i\omega_m)]$$

with

$$\phi^\top(-i\omega_m) = \begin{pmatrix} \delta\Delta(-i\omega_m) & \delta D(-i\omega_m) \end{pmatrix} ,$$

$$M(i\omega_m) = \begin{pmatrix} H^{-1}(i\omega_m) & \chi_{\Delta D}(i\omega_m) \\ \chi_{D\Delta}(i\omega_m) & -\frac{1}{g^2} P^{-1}(i\omega_m) \end{pmatrix} ,$$

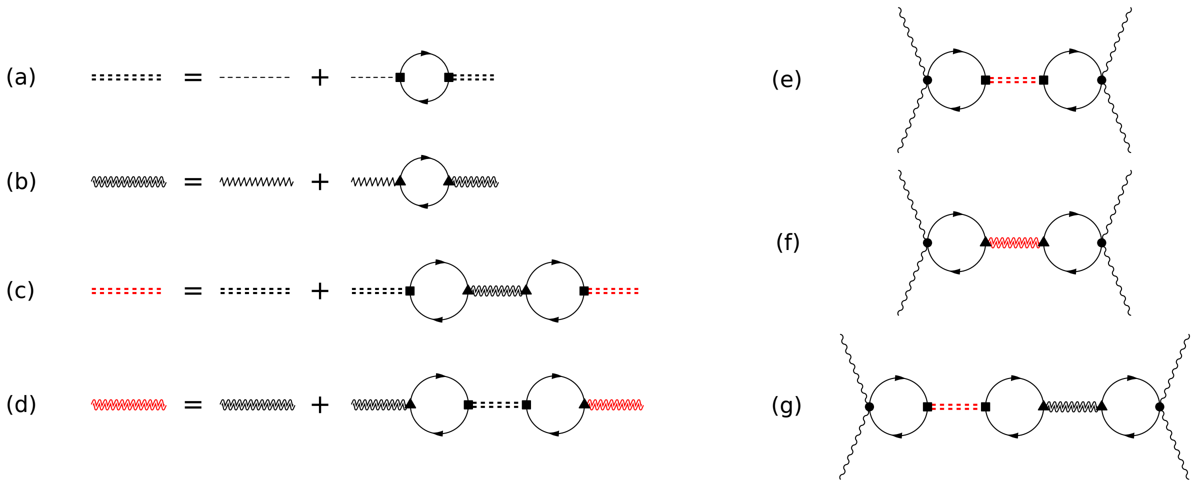

Figure S8 Diagrammatic representation of the terms occuring in the effective action. (a) Higgs propagator defined as the renormalization of the pairing interaction. **(b)** Renormalized phonon propagator due to electron-phonon interaction. **(c)** Renormalized Higgs propagator due to interaction with CDW **(d)** Renormalized phonon propagator due to interaction with Higgs **(e),(f),(g)** Final terms in fourth order action, i.e. **(e)** Higgs, **(f)** CDW, **(g)** mixed contribution.

$$b(i\omega_m) = \begin{pmatrix} -\sum_{ij} \chi^{ij}_{\Delta A^2}(i\omega_m) A^2_{ij}(i\omega_m) \\ -\sum_{ij} \chi^{ij}_{DA^2}(i\omega_m) A^2_{ij}(i\omega_m) \end{pmatrix}.$$

In these expressions, we defined the Higgs progagator as

$$H^{-1}(i\omega_m) = \frac{2}{V} + \chi_{\Delta\Delta}(i\omega_m) = 2\sum_{\boldsymbol{k}} \frac{4\Delta^2 - (i\omega_m)^2}{E_{\boldsymbol{k}}(4E_{\boldsymbol{k}}^2 - (i\omega_m)^2)} \tanh(\beta E_{\boldsymbol{k}}/2)$$

which can be understood as an RPA series renormalization of the pairing interaction as shown diagrammatically in Fig. S8a. Analogously, the renormalized phonon propagator is defined as shown in Fig. S8b. It reads

$$P^{-1}(i\omega_m) = P_0^{-1}(i\omega_m) - g^2\chi_{DD}(i\omega_m) = -\frac{\Omega_{\boldsymbol{Q}}^2 - (i\omega_m)^2}{2\omega_{\boldsymbol{Q}}}$$

with

$$\Omega_{\boldsymbol{Q}}^2 = \omega_{\boldsymbol{Q}}^2 + 2\omega_{\boldsymbol{Q}} g^2 \chi_{DD}(i\omega_m) = \omega_{\boldsymbol{Q}}^2 W \sum_{\boldsymbol{k}} g_{\boldsymbol{k}}^2 \frac{4D_{\boldsymbol{k}}^2 - (i\omega_m)^2}{E_{\boldsymbol{k}}(4E_{\boldsymbol{k}}^2 - (i\omega_m)^2)} \tanh(\beta E_{\boldsymbol{k}}/2)\,.$$

Hereby, the susceptibilities are defined as

$$\mathrm{X}_{\alpha\beta\gamma\delta}(\boldsymbol{k}, i\omega_n) = \frac{1}{\beta}\sum_{i\omega_n} \mathrm{tr}\big[G_0(\boldsymbol{k}, i\omega_n)\, \tau_\alpha \otimes \tau_\beta\, G_0(\boldsymbol{k}, i\omega_m + i\omega_n)\, \tau_\gamma \otimes \tau_\delta\big]\,,$$

$$\chi_{\Delta\Delta}(i\omega_m) = \sum_{\boldsymbol{k}} \mathrm{X}_{1010} = -8\sum_{\boldsymbol{k}} \frac{D_{\boldsymbol{k}}^2 + \epsilon_{\boldsymbol{k}}^2}{E_{\boldsymbol{k}}(4E_{\boldsymbol{k}}^2 - (i\omega_m)^2)} \tanh(\beta E_{\boldsymbol{k}}/2)\ ,$$

$$\chi_{\Delta\mathrm{D}}(i\omega_m) = \sum_{\boldsymbol{k}} g_{\boldsymbol{k}} \mathrm{X}_{1031} = 8\sum_{\boldsymbol{k}} g_{\boldsymbol{k}} \frac{\Delta D_{\boldsymbol{k}}}{E_{\boldsymbol{k}}(4E_{\boldsymbol{k}}^2 - (i\omega_m)^2)} \tanh(\beta E_{\boldsymbol{k}}/2)\ ,$$

$$\chi^{ij}_{\Delta\mathrm{A}^2}(i\omega_m) = \sum_{\boldsymbol{k}} \frac{1}{2}\partial^2_{ij}\epsilon_{\boldsymbol{k}} \mathrm{X}_{1033} = -4\sum_{\boldsymbol{k}} \partial^2_{ij}\epsilon_{\boldsymbol{k}} \frac{\epsilon_{\boldsymbol{k}}\Delta}{E_{\boldsymbol{k}}(4E_{\boldsymbol{k}}^2 - (i\omega_m)^2)} \tanh(\beta E_{\boldsymbol{k}}/2)\ ,$$

$$\chi_{\mathrm{DD}}(i\omega_m) = \sum_{\boldsymbol{k}} g_{\boldsymbol{k}}^2 \mathrm{X}_{3131} = -8\sum_{\boldsymbol{k}} g_{\boldsymbol{k}}^2 \frac{\Delta^2 + \epsilon_{\boldsymbol{k}}^2}{E_{\boldsymbol{k}}(4E_{\boldsymbol{k}}^2 - (i\omega_m)^2)} \tanh(\beta E_{\boldsymbol{k}}/2)\ ,$$

$$\chi^{ij}_{\mathrm{DA}^2}(i\omega_m) = \sum_{\boldsymbol{k}} g_{\boldsymbol{k}} \frac{1}{2}\partial^2_{ij}\epsilon_{\boldsymbol{k}} \mathrm{X}_{3133} = -4\sum_{\boldsymbol{k}} g_{\boldsymbol{k}}\, \partial^2_{ij}\epsilon_{\boldsymbol{k}} \frac{\epsilon_{\boldsymbol{k}} D_{\boldsymbol{k}}}{E_{\boldsymbol{k}}(4E_{\boldsymbol{k}}^2 - (i\omega_m)^2)} \tanh(\beta E_{\boldsymbol{k}}/2)\ ,$$

with the symmetry property $\mathrm{X}_{\gamma\delta\alpha\beta}(\boldsymbol{k}, i\omega_m) = \mathrm{X}_{\alpha\beta\gamma\delta}(\boldsymbol{k}, -i\omega_m)$. Finally, we integrate out the amplitude fluctuations obtaining $S^{(4)} \propto b^\top M^{-1} b$ with the inverse of M

$$M^{-1}(i\omega_m) = \begin{pmatrix} \widetilde{H}(i\omega_m) & g^2\chi_{\Delta D}(i\omega_m)P(i\omega_m)\widetilde{H}(i\omega_m) \\ g^2\chi_{D\Delta}(i\omega_m)\tilde{P}(i\omega_m)H(i\omega_m) & -g^2\tilde{P}(i\omega_m) \end{pmatrix}$$

where we identify the renormalized Higgs and phonon propagator

$$\widetilde{H}(i\omega_m) = \frac{1}{H^{-1}(i\omega_m) + g^2\chi_{\Delta D}(i\omega_m)\chi_{D\Delta}(i\omega_m)P(i\omega_m)}$$

$$\tilde{P}(i\omega_m) = \frac{1}{P^{-1}(i\omega_m) + g^2\chi_{\Delta D}(i\omega_m)\chi_{D\Delta}(i\omega_m)H(i\omega_m)}$$

which can be again understood as an RPA renormalization as shown in Fig. S8c-d. For the further analysis, we assume linear polarized light in x-direction, i.e.

$$A(t) = \begin{pmatrix} A_0\cos(\Omega t) \\ 0 \end{pmatrix},$$

$$A^2(\omega) = A_0^2\left(\delta(\omega) + \frac{1}{2}\delta(\omega - 2\Omega) + \frac{1}{2}\delta(\omega + 2\Omega)\right)\ .$$

As a result, only the derivative $\partial^2_{xx}\epsilon_{\boldsymbol{k}}$ remains in the respective susceptibilities such that we neglect the polarization indices in the following. With this, the fourth order action finally reads after analytic continuation $i\omega_m \to \omega + i0^+$

$$S^{(4)} = \frac{1}{2}\int \mathrm{d}\omega K^{(4)}(\omega)A^2(\omega)A^2(-\omega)$$

where the kernel is given by

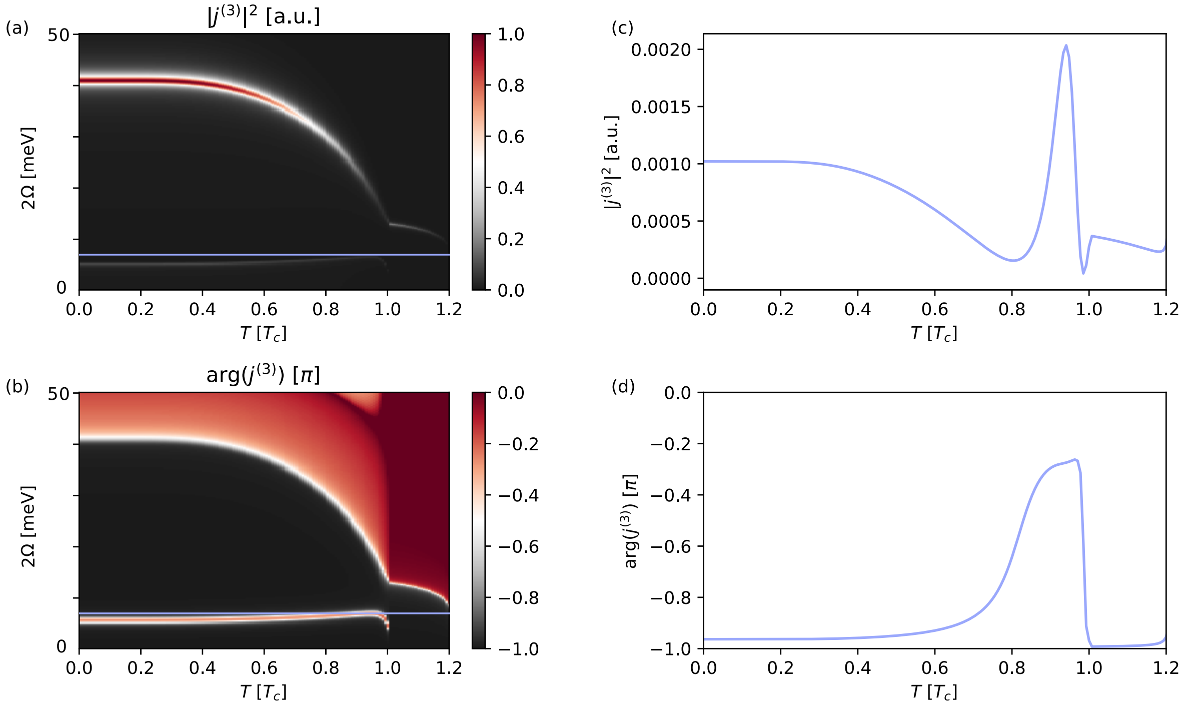

Figure S9 THG signal as function of frequency and temperature. (a) Intensity and **(b)** phase of THG signal as a function of temperature and **driven frequency** (2Ω). **(c)** THG intensity and **(d)** THG phase as function of temperature for a fixed driven frequency of ~ 6 meV (1.5 THz), which correspond to the horizontal line cuts in **(a)** and **(b)**.

$$K^{(4)}(\omega) = -\chi_{A^2\Delta}(\omega)^2\widetilde{H}(\omega) - g^2\chi_{A^2D}(\omega)^2\widetilde{P}(\omega) + 2g^2\chi_{A^2\Delta}(\omega)\chi_{A^2D}(\omega)\chi_{\Delta D}(\omega)\widetilde{H}(\omega)P(\omega)\,.$$

The three contributions are related to the Higgs excitation, CDW excitation and a mixed term which are shown in Fig. S8e-g. As a result, the induced THG current is directly proportional to the fourth order kernel

$$j^{(3)}(3\Omega) = -\frac{\delta S^{(4)}}{\delta A_\alpha(-\omega)}\bigg|_{3\Omega} \propto K^{(4)}(2\Omega)$$

We evaluate the three diagrams using the parameters Δ = 20 meV, D = 10 meV, ω_0 = 20 meV, t = 100 meV, and a broadening $\Omega + i\eta$ with η = 0.1 meV on a 2d momentum grid with 2000 × 2000 points for varying driving frequency and temperature. Hereby, the superconducting gap $\Delta(T)$ and CDW gap $D(T)$ is self-consistently evaluated for each temperature by iteratively solving the two gap equations simultaneously. It is important to emphasize that this model does not quantitatively describe the experiment on cuprates but serves as a general proof of principle for the interplay between the Higgs mode and a CDW fluctuation. As one can see in Fig. S9, two resonances appear

in the THG spectrum corresponding to the renormalized Higgs mode at ~ 40 meV and at the renormalized phonon frequency at ~ 6 meV at $T = 0$. The temperature dependence roughly follows the temperature dependence of the order parameter. Each resonance is accompanied by a positive phase change of roughly π slightly reduced due to the broadening. The phase signature is more clear than the peak in the amplitude for the lower mode. In addition, the interference between the two modes is visible by an antiresonance characterized by a negative phase change of roughly π close to the lower mode. As one can see, a horizontal cut in the 2d plot near the lower mode shows the negative phase change as observed in the experiment. The exact shape of the horizontal cuts depend on the details of the model, yet the antiresonance behavior with a negative phase change is always visible as long as the driving frequency matches the energy of antiresonance for some temperature.

S11. Evidence for anti-resonance above T_c

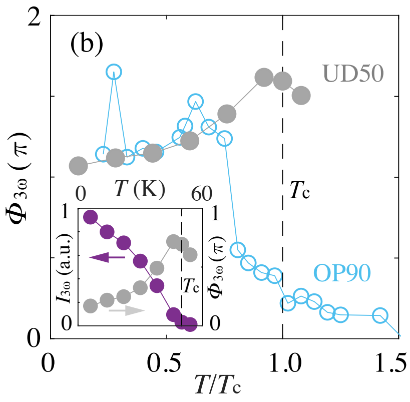

Figure S10 THG from bilayer cuprates THG phase response of an underdoped $DyBa_2Cu_2O_{7-x}$ (UD50) measured with 0.5 THz driving frequency, superposed on top of the THG phase response of an optimally doped $DyBa_2Cu_2O_{7-x}$(OP90) measured with 0.7 THz driving frequency as reported in ref(3). Inset shows the temperature dependence of THG intensity and phase of the UD50 sample. $\Phi_{3\omega}$ cannot be reliably extracted beyond T_c as the THG signal becomes very low there, due in part to the use of a table-top THz source for this measurement. It can be seen from the main figure that an anti-resonance seems to appear just above T_c.

We measured the THG response of an underdoped $DyBa_2Cu_2O_{7-x}$(UD50) film with 0.5 THz driving frequency. A positive evolution of the THG phase is observed below T_c with the start of a downturn right at T_c, indicating a potential anti-resonance above T_c. We compare the results of this UD50 sample to an optimally doped $DyBa_2Cu_2O_{7-x}$(OP90) sample measured previously with 0.7 THz driving frequency. The anti-resonance in that case takes place significantly below T_c.

References

1. B. Green. , S. Kovalev, V. Asgekar, G. Geloni, U. Lehnert, T. Golz, M. Kuntzsch, C. Bauer, J. Hauser, J. Voigtlaender, B. Wustmann, I. Koesterke, M. Schwarz, M. Freitag, A. Arnold, J. Teichert, M. Justus, W. Seidel, C. Ilgner, N. Awari, D. Nicoletti, S. Kaiser, Y. Laplace, S. Rajasekaran, L. Zhang, S. Winnerl, H. Schneider, G. Schay, I. Lorincz, A. A. Rauscher, I. Radu, S. Mährlein, T. H. Kim, J. S. Lee, T. Kampfrath, S. Wall, J. Heberle, A. Malnasi-Csizmadia, A. Steiger, A. S. Müller, M. Helm, U. Schramm, T. Cowan, P. Michel, A. Cavalleri, A. S. Fisher, N. Stojanovic, M. Gensch. High-Field High-Repetition-Rate Sources for the Coherent THz Control of Matter. *Sci. Rep.* **6**, 22256 (2016).

2. S. Kovalev, B. Green, T. Golz, S. Maehrlein, N. Stojanovic, A. S. Fisher, T. Kampfrath, M. Gensch. Probing ultra-fast processes with high dynamic range at 4th-generation light sources: Arrival time and intensity binning at unprecedented repetition rates. *Structural Dynamics* **4**, 024301 (2017).

3. H. Chu, M.-J. Kim, K. Katsumi, S. Kovalev, R. D. Dawson, L. Schwarz, N. Yoshikawa, G. Kim, D. Putzky, Z. Z. Li, H. Raffy, S. Germanskiy, J.-C. Deinert, N. Awari, I. Ilyakov, B. Green, M. Chen, M. Bawatna, G. Cristiani, G. Logvenov, Y. Gallais, A. V. Boris, B. Keimer, A. P. Schnyder, D. Manske, M. Gensch, Z. Wang, R. Shimano, S. Kaiser. Phase-resolved Higgs response in superconducting cuprates. *Nat. Commun.* **11** 1793 (2020).

4. G. Kim, G. Christiani, G. Logvenov, S. Choi, H.-H. Kim, M. Minola, B. Keimer. Selective formation of apical oxygen vacancies in $La_{2-x}Sr_xCuO_4$. *Phys. Rev. Mat.* **1**, 054801 (2017).

5. K. Katsumi, N. Tsuji, Y. I. Hamada, R. Matsunaga, J. Schneeloch, R. D. Zhong, G. D. Gu, H. Aoki, Y. Gallais, R. Shimano. Higgs Mode in the d-Wave Superconductor $Bi_2Sr_2CaCu_2O_{8+x}$ Driven by an Intense Terahertz Pulse. *Phys. Rev. Lett.* **120**, 117001 (2018).

6. R. Matsunaga, N. Tsuji, H. Fujita, A. Sugioka, K. Makise, Y. Uzawa, H. Terai, Z. Wang, H. Aoki, R. Shimano. Light-induced collective pseudospin precession resonating with Higgs mode in a superconductor. *Science* **345**, 1145-1149 (2014).

7. N. Tsuji, H. Aoki. Theory of Anderson pseudospin resonance with Higgs mode in superconductors. *Phys. Rev. B* **92**, 064508 (2015).

8. N. Tsuji, Y. Murakami, H. Aoki. Nonlinear light–Higgs coupling in superconductors beyond BCS: Effects of the retarded phonon-mediated interaction. *Phys. Rev. B* **94**, 224519 (2016).

9. L. Schwarz, D. Manske. Theory of driven Higgs oscillations and third-harmonic generation in unconventional superconductors. *Phys. Rev. B* **101**, 184519 (2020).

10. T. Cea, C. Castellani, L. Benfatto. Nonlinear optical effects and third-harmonic generation in superconductors: Cooper pairs versus Higgs mode contribution. *Phys. Rev. B* **93**, 180507(R) (2016).

11. G. Seibold, M. Udina, C. Castellani, L. Benfatto. Third harmonic generation from collective modes in disordered superconductors. *Phys. Rev. B* **103**, 014512 (2021).

12. F. Gabriele, M. Udina, L. Benfatto. Non-linear Terahertz driving of plasma waves in layered cuprates. *Nat. Commun.* **12**, 752 (2021).

13. N. Tsuji, Y. Nomura. Higgs-mode resonance in third harmonic generation in NbN superconductors: Multiband electron-phonon coupling, impurity scattering, and polarization-angle dependence. *Phys. Rev. Res.* **2**, 043029 (2020).

14. M. Silaev. Nonlinear electromagnetic response and Higgs-mode excitation in BCS superconductors with impurities. *Phys. Rev. B* **99**, 224511 (2019).

15. Y. Murotani, R. Shimano. Nonlinear optical response of collective modes in multiband superconductors assisted by nonmagnetic impurities. *Phys. Rev. B* **99**, 224510 (2019).

16. R. Haenel, P. Froese, D. Manske, L. Schwarz. Time-resolved optical conductivity and Higgs oscillations in two-band dirty superconductors. *Phys. Rev. B* **104**, 134504 (2021).

17. M. Puviani, A. Baum, S. Ono, Y. Ando, R. Hackl, D. Manske. Calculation of an enhanced A_{1g} symmetry mode induced by Higgs oscillations in the Raman spectrum of high-temperature cuprate superconductors. *Phys. Rev. Lett.* **127**, 197001 (2021).

18. T. Cea, P. Barone, C. Castellani, L. Benfatto. Polarization dependence of the third-harmonic generation in multiband superconductors. *Phys. Rev. B* **97**, 094516 (2018).

19. D. H. Torchinsky, H. Chu, L. Zhao, N. B. Perkins, Y. Sizyuk, T. Qi, G. Cao, D. Hsieh. Structural Distortion-Induced Magnetoelastic Locking in Sr_2IrO_4 Revealed through Nonlinear Optical Harmonic Generation. *Phys. Rev. Lett.* **114**, 096404 (2015).

20. M. Fujita, H. Hiraka, M. Matsuda, M. Matsuura, J. M. Tranquada, S. Wakimoto, G. Xu, K. Yamada. Progress in Neutron Scattering Studies of Spin Excitations in High-T_c Cuprates. *J. Phys. Soc. Jpn.* **81**, 011007 (2012).

21. B. Lake, G. Aeppli, K. N. Clausen, D. F. McMorrow, K. Lefmann, N. E. Hussey, N. Mangkorntong, M. Nohara, H. Takagi, T. E. Mason, A. Schroder. Spins in the Vortices of a High-Temperature Superconductor. *Science* **291**, 1759-1762 (2001).

22. M. Iizawa, S. Kosugi, F. Koike, Y. Azuma The quantum and classical Fano parameter q. *Phys. Scr.* **96** 055401 (2021).

23. T. Cea, L. Benfatto. Nature and Raman signatures of the Higgs amplitude mode in the coexisting superconducting and charge-density-wave state. *Phys. Rev. B* **90**, 224515 (2014).
